# Mind, body, and spirit: a constructivist grounded theory study of wellness among middle-class Black women

**DOI:** 10.1080/17482631.2023.2278288

**Published:** 2023-11-18

**Authors:** Quenette L. Walton, Jacquelyn V. Coats, Kia Skrine Jeffers, Joan M. Blakey, Alexandra N. Hood, Tyreasa Washington

**Affiliations:** a Graduate College of Social Work, University of Houston, Houston, TX, USA; b Brown School of Social Work, Washington University in St. Louis, St. Louis, MO, USA; c School of Nursing, Center for the Study of Racism, Social Justice & Health, Fielding School of Public Health, University of California, Los Angeles, CA, USA; d School of Social Work, University of Minnesota, St. Paul, MN, USA; e Department of Social Work, University of North Carolina at Greensboro, Greensboro, NC, USA, and Child Trends, Bethesda, MD, USA

**Keywords:** Black women, wellness, constructivist grounded theory, intersectionality, cultural perspectives

## Abstract

Previous studies show that Black women in the United States experience disproportionately poorer health outcomes compared to women of other racial/ethnic groups. Recently the focus is on improving the health of Black women in the United States. However, there is little empirical evidence on what Black women need to improve their health to be well. The goal of this constructivist grounded theory was to increase the understanding of wellness among middle-class Black women (*N* = 30) in a large Midwestern city in the United States through an intersectional lens. The findings show that the connection and balance between mind, body, and spirit was the core experience of wellness among middle-class Black women. Mind, body, and spirit was described in three ways—(a) mentally managing, (b) physically caring for my body, and (c) connecting spiritually—with the women also noting the barriers and facilitators they endured to be well. Each of these categories highlight the tension middle-class Black women experience with trying to be well. Implications for future practice and research with middle-class Black women are discussed.

Black women in the United States experience disproportionately poorer health outcomes compared to women of other racial/ethnic groups, including shorter life expectancies, higher rates of maternal mortality, breast cancer mortality, obesity, and preventable chronic conditions such as hypertension and type 2 diabetes (Chinn et al., 2021; Williams, 2002). These racial-gender patterns of health disparities persist after controlling for socioeconomic status (SES), and at times may even be higher for Black women at higher SES levels (Cummings & Braboy Jackson, 2008). For example, Black women of greater SES levels are more likely to have higher rates of preterm births, higher infant mortality rates, and more likely to die while pregnant than lower SES White women (Adelman et al., 2008; Chinn et al., 2021; Williams, 2002; Braveman et al., 2015). Allostatic load, an indicator of accelerating ageing, has also been found to be higher among nonpoor Black women compared to poor White women (Geronimus et al., 2006). These well-documented health inequities indicate a need to better understand health practices and strategies that enhance or impede wellness for Black women with different SES and using an intersectional lens.

Most wellness models have been developed from the perspectives and experiences of White Americans. These models often fail to capture wellness among diverse groups or consider other critical cultural and contextual factors (e.g., race, class, and gender) relevant to Black women’s lives in the United States (Assari, 2018; Gamby et al., 2021). Relying on Western theories and practices of health and wellness often is incomplete and can be oppressive towards Black women (French et al., 2020). Moreover, if considering Black women, and middle-class Black women more specifically, it is essential to recognize that their lived experiences and experiences with wellness are shaped by a multitude of sociocultural factors (e.g., religion, health practices, social values, norms, geographic location) unique to their race, class, and gender identities (Chinn et al., 2021). Thus, the current study sought to understand middle-class Black women’s experiences with wellness through an intersectional lens. Because Black women are essential to the Black family and community, it is critical that we understand wellness among Black women, the factors that impact their ability to be well, and identify strategies that enable them to enhance their wellness.

## Literature review

While wellness has been the topic of research since the 1960s, there is very little consensus regarding its definition (Roscoe, 2009). Most studies define wellness as the absence of disease or illness (Dunn, 1977). In 1967, the World Health Organization (WHO) defined wellness as more than the absence of illness. In 2020, WHO defined health as “a state of complete physical, mental, and social well-being and not merely the absence of disease or infirmity” (p.1). Dunn (1977) defined wellness as “an integrated method of functioning, which is oriented toward maximizing the potential of which the individual is capable. It requires that the individual maintain a continuum of balance and purposeful direction within the environment where he is functioning” (Dunn, 1977, p. 4).

Despite the fact there is no singular definition of wellness, there are some consistencies across definitions (Roscoe, 2009). Wellness consists of several dimensions that include emotional well-being (i.e., ability to cope with life and create satisfying relationships) and physical well-being (i.e., physical activity, healthy foods, and sleep; National Center for Complementary and Integrative Health, 2018; World Health Organization, 2020). Wellness is believed to be on a continuum as opposed to a finite destination (Dunn, 1977; Lafferty, 1979; Sechrist, 1979).

### Theoretical conceptions of wellness

Theoretical orientations and conceptualizations of wellness have often used three highly favourable models: the biopsychosocial model (Engel, 1977), the six dimensions of wellness model (Hettler, 1980, 1984), and the biopsychosocial-spiritual model (Sulmasy, 2002). The biopsychosocial model has been widely adopted and promoted as a framework acknowledging individuals personal characteristics, surrounding environment, and the various ways biological, psychological, and social factors impact health (Engel, 1977). The six dimensions of wellness model focuses on the integration of the following dimensions: emotional, occupational, physical, social, intellectual, and spiritual (Hettler, 1980, 1984) Alternatively, the biopsychosocial-spiritual model centres the holistic needs of a client by assessing them in four domains of their life—biological, psychological, social, and spiritual—and recognizing that these factors are not mutually exclusive (Sulmasy, 2002).

Despite overall acceptance of the biopsychosocial model, the six dimensions of wellness model, and the biopsychosocial-spiritual model, the potential application of these models of wellness with middle-class Black women is not without challenges. These models lack important considerations and attention to the diverse ways wellness may be experienced and expressed across members of different groups and ignore the sociocultural variations such as religion, health practices, social values, norms, geographic location, in the conceptualizations of wellness. We highlight important contexts related to race, class, and gender when understanding wellness among Black middle-class women because intersectional research that focuses on class differences are needed to better understand how and why class influences differential wellness experiences among Black women (Howell, 2023).

### Black women and wellness

In the past two decades, research has primarily focused on physical wellness among Black women, often investigating issues related to food (Rowe, 2010), obesity, physical activity (Chithambo & Huey, 2013; Dodgen & Spence-Almageur, 2017; Ray, 2014), and chronic disease prevention (Bernstein et al., 2014). Within the same time span, several studies have focused on mental wellness among Black women, specifically as it relates to depression (Donovan & West, 2015; Nicolaidis et al., 2013; Waite & Killian, 2008) or coping with stressors (Jones, 2004; Jones et al., 2014; Woods-Giscombé & Black, 2010). Although these areas of research have highlighted health disparities Black women face, there is a notable gap in the knowledge base of a multidimensional wellness model for Black women that moves beyond physical or mental health and socioeconomic status (Howell, 2023; Obi et al., 2023).

Although understudied, a few researchers have endeavoured to understand how Black women experience and express wellness. The work of Jones et al. (2015) found holistic wellness for Black women receiving mental health or substance abuse treatment centred on emotional, physical, and spiritual wellness. Bell (2017) found that Black women mental health experts conceptualized inner peace including dimensions of body, mind, spirit, social, economic, and political balance. Congruent with other findings, Black Women’s Health Imperative (BWHI, 2019) observed that healthy Black women prioritized their emotional health, followed by financial health, and then physical health. Despite these compatible findings, one limitation is that each study discussed Black women as a monolith. Therefore, it is important to tease out how experiences with wellness are shaped by sociocultural factors as well as intersectional identities among Black women in general, but middle-class Black women in particular.

Along these same lines, the importance of faith, religion, and prayer in the lives of Black women have been widely documented, finding it to be associated with greater life satisfaction and health outcomes (Utsey et al., 2007; Spates et al., 2020, Newlin, 2002; Mitchem, 2002; Mattis, 2000; Heath, 2006; Thomas et al., 2008). Other scholars have identified that Black women want issues of racism and discrimination to be recognized and addressed as a part of their wellness experiences (Jones et al., 2015; Nicolaidis et al., 2013). Still other research findings have recognized the importance of indigenous beliefs and values that emphasize healing and wellness such as the NTU psychotherapy model (French et al., 2020; Woods-Giscombé & Black, 2010). The NTU psychotherapy model has been highlighted as a cultural framework that utilizes Afrocentric principles, emphasizes the unity of mind, body, and spirit, and is a particularly useful mind-body intervention with Black women (Woods-Giscombé & Black, 2010). The NTU psychotherapy model is based on principles that centre collectivism, as well as addresses and acknowledges the historical and current oppression experienced by Black women (Woods-Giscombé & Black, 2010). Overall, these findings suggest Black women proactively engage in wellness practices; yet there is still a paucity of research about Black women’s experiences with wellness. Thus, an important distinction to make is that it is not just about understanding Black women’s experiences with wellness, but rather the context in which they live and the “social position, processes of oppression or privilege, policies or institutional practices” (Bauer, 2014, p. 10), and assumptions of wellness among middle-class Black women specifically.

### Race, class, and gender factors influencing wellness among Black women

The intersection of race, class, and gender often affect middle class Black women’s ability to be well (Oliver et al., 2019; Sacks, 2019). A few studies have examined this intersection. Lewis and Neville (2015) constructed the Gendered Racial Microaggression Scale (GRMS), using an intersectional framework, to appraise stress levels and frequency of gendered racism microaggressions due to the lack of scales measuring such experiences. Although framed as a limitation, Lewis and Neville’s (2015) sample (*N* = 265) was comprised mostly of self-identified middle-class Black women (55%), thus lending itself well to this paper. In the validation phase of the scale construction, they found that over 90% of Black women had experienced gendered racism, which increased their levels of psychological distress. Four themes of gendered racism microaggressions were found including (a) assumptions of beauty and sexual objectification, (b) silenced and marginalized, (c) Strong Black Women stereotypes, and (d) Angry Black Women stereotypes (Lewis & Neville, 2015).

Many of the settings in which the Black women in this study were discriminated against revolved around professional settings. Similarly, in a follow-up study utilizing the GRMS, with a sample of 60% self-identified middle-class Black women (*N* = 231), Lewis and colleagues (Lewis et al., 2017) found that the more gendered racial microaggressions one experienced the more negative mental health outcomes they experienced. However, having a positive gendered racial identity offered partial support in mitigating the impact of microaggressions on mental health outcomes (Lewis et al., 2017). Although not discussed within their findings and given their large samples that identified as Black middle-class (55% & 65%, respectively), results of both these studies may suggest that class does not provide a protective factor to the harmful experiences of gendered racism and Black women’s well-being and wellness practices.

Another way race, class, and gender have intersected to impact wellness among Black women has focused on the effects of gendered racism on psychological distress and the role of Africultural coping style (Thomas et al., 2008). Africultural coping styles were determined through the *Africultural Coping Styles Inventory* (ACSI; Thomas et al., 2008). According to Thomas and colleagues (Thomas et al., 2008), ACSI measures coping skills that are specific to Black culture and includes cognitive/emotional debriefing (e.g., thought avoidance), spiritual-centred coping (e.g., spirituality), collective coping (e.g., social support), and ritual-centred coping (e.g., praying to a deity). In their sample of 344 Black women, nearly 65% of respondents reported an income that falls within the middle-class income level. Almost all of the women reported gendered racism discrimination, mostly in interpersonal relationships and professional settings, which had a persistent negative effect on the psychological distress of Black women. Thomas and colleagues (Thomas et al., 2008) found a significant positive relationship between psychological distress and gendered racism, although this was found to be partially mediated by one’s Africultural coping style. That is, cognitive-emotional debriefing was found to support Black women in the harmful impact on their psyche due to gendered racism. However, the partial mediation suggests that there are other coping skills being utilized to support Black women’s wellness, thus highlighting the need for the current study.

Lastly, studies have illustrated that many upwardly mobile, middle-class Black women face numerous stressors, including a sense of responsibility to support their networks, pressures to positively represent other Black people and challenge negative stereotypes, and experiences of ongoing racial bias and discrimination in settings such as healthcare and workplaces that may hinder their experiences with wellness (Hudson et al., 2020, 2023; Nelson et al., 2016; Sacks, 2017; Walton & Boone, 2019). For instance, Walton and Boone (2019), sought to contextualize the experiences of middle-class Black women and the role race, class, and gender may have on their experiences. Using intersectionality, person-in-environment, and cultural frameworks, the authors interviewed 30 middle-class Black women to thematically analyse their experiences. An overarching expression of feeling unheard was supported by three themes which noted that they felt Black women’s depression was rarely discussed, Black women were unable to be vulnerable, and that their intersectional social identities (as it related to family, community, and caregiving) were further complicated by their upward mobility. The women in this study noted that they were financially supporting their less financially stable family members, which resulted in depression. Thus, Walton and Boone (2019) suggest that depression is often linked to having intersecting identities. The authors also posit that experiences of depression for middle-class Black women is often due to the pressure of being a caregiver to less financially stable family members, which adversely influence their mental health Walton and Boone (2019). Research rarely discusses the experiences of Black middle-class women and their health outcomes; thus, Walton and Boone’s (2019) study highlight the importance of centring their experiences to understand how to better support their overall well-being.

### Middle-class Black women and wellness

The relationship between wellness and race, class, and gender have not been adequately studied, but it likely reflects that many of the studies pathologizes Black women’s lived experiences and treat race as a proxy for class when explaining adverse health outcomes among Black women (Howell, 2023; Sacks, 2019). However, despite their economic gains, middle-class Black women are disproportionately sicker, die earlier, and fare worse than White women with less education across all health outcomes (Sacks, 2019). Thus, Black middle-class women are vulnerable to adverse health outcomes and stressors which may influence their perceptions and experiences of wellness because they often have to navigate multiple oppressions (Sacks, 2019). For instance, gendered racism and classism may place middle-class Black women at risk for pregnancy complications (Bridges, 2020) and mental health issues like depression (Lewis & Neville, 2015; Lewis et al., 2017), which can increase stress, limit work opportunities, and create challenges for them to seek treatment. Additionally, due to gendered racism, middle-class Black women often endure stereotypes based on Black womanhood which also have an influence on their mental health (Lewis & Neville, 2015). As such understanding wellness among middle-class Black women through an intersectional lens could increase opportunities for this group of women to begin to address the disproportionately poorer health outcomes they experience.

### Theoretical framework: intersectionality

Intersectionality is the theoretical approach guiding this study. In social work, psychology, and other fields of study, intersectionality has been commended because this approach (a) allows researchers flexibility to centre the multiple identities of Black women, (b) stresses forms of oppression are not additive, and (c) does not limit the focus of one identity at the expense of another (Crenshaw, 1991; Jordan-Zachery, 2007). Because intersectionality emphasizes interlocking oppressions experienced by Black women, it allows researchers to treat social identities as relational (Collins, 2000; Crenshaw, 1991). Further, intersectionality emphasizes the importance of making Black women visible within larger society to humanize their experiences at the intersection of their identities (Hancock, 2016; Jordan-Zachery, 2007). As such, we sought to understand middle-class Black women’s experiences with wellness given their multiple interesting identities and the salience of these identities in the context of their wellness practices. Because gender is always raced, race is always gendered, and these identities are shaped by the context of the other identity, such as class (Walton & Oyewuwo-Gassikia, 2017), it is critical to qualitatively understand the combined impact of racism, classism, and sexism to better understand wellness experiences among middle-class Black women.

### Current study

Black women experience a significant amount of stress from race and gender-based discrimination (Howell, 2023; Spates et al., 2020), which consequently influence their wellness experiences. Additionally, literature has extensively assumed Black women are a monolithic group and middle-class Black women would inevitably share similar experiences to low-income Black women (Sacks, 2017, 2019). Further, studies have shown Black women who identify as middle-class may experience additional strain due to familial and personal challenges and must contend with their class status’s simultaneous privilege and marginalization (Hudson et al., 2020; Sacks, 2017, 2019). Because the current literature lacks an inclusive model to understand wellness among middle-class Black women, we used constructivist grounded theory (Charmaz, 2014) to develop a new model. For this study, we examined the following research questions: (a) How do middle-class Black women experience wellness? (b) What factors influence middle-class Black women’s experiences with wellness, paying particular attention to their race, class, and gender identities to understand how wellness is perceived?

## Method

### Recruitment and participants

This study examined 30 middle-class Black women’s (See Table I for participant demographics) experiences with wellness and how their experiences of wellness was influenced by their race, class, and gender identities. Participants were eligible to participate in this study if they (a) self-identified as Black and female, (b) self-reported middle-class status based on educational level, income, and occupation status; and (c) self-reported experiences with wellness. Participants were recruited via several methods: (a) social media outlets, (e.g., Facebook), (b) emailed flyers to predominantly Black female sororities, and (c) posted flyers in predominantly Black businesses in Chicago, Illinois.

**Table I.** Additional demographic data of participants.

Participant pseudonym	Age	Relationship status	Educational level (By degree)	Profession
Essie	35	Married	Master’s	Social worker/community activist
Elaine	45	Single	Master’s	Field training police officer/graduate student
Kendra	35	Single	Master’s	Professor
Shelia	39	Single	Bachelor’s	Administrator in president’s office
Lisa	33	Married	Master’s	Executive assistant & executive director of own company
Jennifer	37	Married	Master’s	Researcher
Danielle	32	Married	Bachelor’s	Account manager supervisor/business owner
Lillie	33	Single	Master’s	Social worker
Katie	44	Married	Master’s	Editor/writer
Valerie	39	Married	Doctorate	Professor
Pink	39	Married	JD	Attorney
Indigo	34	In a committed relationship	Master’s	Intercultural trainer & consultant
Stacey	32	Married	Master’s	IT project manager
Nightbird	44	Divorced	Master’s	Clinical social worker/administrator
Coral	42	Divorced	Bachelor’s	Sales manager
Keisha	39	Married	Bachelor’s	Community activist/graduate student
Queen	37	Married	Master’s	Registered nurse
Sherry	45	In a committed relationship	Doctorate	Evaluator
Jade	37	Married	Master’s	Clinical social worker/manager
Afro	40	Single	Master’s	Marketing
Roxanne	31	Married	Doctorate	Assistant professor
Monique	35	Single	Master’s	Social worker/therapist
Angela	37	Married	Doctorate	Researcher
Karen	32	Married	Bachelor’s	Teacher
Emily	42	In a committed relationship	Bachelor’s	Law enforcement
Zoe	35	Married	Doctorate	Educator
Michelle	43	Single	Doctorate	Dentist
Lola	40	In a committed relationship	Bachelor’s	Project manager
Daphne	30	Married	Doctorate	Assistant professor
Amber	44	Married	Master’s	Research assistant/graduate student

*Note*. (*N* = 30); All demographic data are self-reported.

There is little agreement on overall criteria for defining the Black middle class. For empirical identification, scholars have marked off a segment of the population above a specific bound with respect to education, occupation, and income (Darity et al., 2020; Lacy, 2007). For this study, we used the same bound with respect to education, occupational status, and income to determine the women’s middle-class status. However, the minimum income noted to confirm middle-class status for this study differed from what Lacy (2007) identified as middle-class income (i.e., $50,000) because participants lived in a city where the median salary earnings at the time of this study for Blacks with a bachelor’s degree or higher was $40,000 or greater for an individual or $60,000 or greater for a household (Joint Center for Political and Economic Studies Health Policy Institute, 2012). Earning incomes at the individual and household level of $40,000 or $60,000, respectively, placed Blacks securely within middle-class status within the city of Chicago (Joint Center for Political and Economic Studies Health Policy Institute, 2012). However, it should be noted that for Black women, the individual and household incomes outlined above have persisted for the past 50 years and is noted at every education level, even when graduate degrees have been earned (Pew Research Center, 2023; Tucker, 2019).Thus, participants were considered middle-class for this study if they had a bachelor’s degree or higher; earned at least $40,000 U.S. dollars per year; and had a professional, managerial, technical, or administrative job.

A total of 30 women participated in this study. Participants’ ages ranged between 30 and 45 with a mean age of 37.66. Of the 30 women who participated in this study 57% were married (*n* = 17), 36% (11) were single or in committed relationships, 6% (*n* = 2) were divorced, and 60% (*n* = 18) had children. In terms of education, 23% (*n* = 7) earned a bachelor’s degree, half of the women earned a master’s degree (*n* = 15), and 26% (*n* = 8) earned a doctorate. Last, more than half 63% (*n* = 19) of the women grew up in a middle-class home and women earned between $40,000 and $235,000 U.S. dollars (see Table I for additional demographic data).

### Research team

In alignment with the constructivist approach of grounded theory, the positionality of the researcher is important (Charmaz, 2006, 2014). Within the constructivist approach, researchers are seen as co-creators of the data who have biases and assumptions regarding the data that could influence their interpretation (Charmaz, 2014). Therefore, it is important for researchers employing a grounded theory approach to be reflective of how their positions, preconceptions, backgrounds, and lived experiences influence the study (Charmaz, 2014; Pearson, 2004).

All the authors of this study identified as middle-class Black women. The first author is an assistant professor at a research-intensive university and a licenced clinician with prior experience working with Black women of diverse ages and socioeconomic statuses. She also has experience conducting qualitative studies and analysing qualitative data. For this study, the first author was responsible for recruiting and interviewing participants, analysing data, and finalizing major categories from the data. The second author is a doctoral student with experience examining health disparities among Black women and identifying protective factors for health. She was responsible for the literature review, as well as assisting with finalizing categories and the results. The third author is an assistant professor in a college of nursing at a research-intensive institution on the West Coast. Because of her expertise with identifying how race, class, and gender intersects and influences mental health outcomes among Black women, she assisted with data analysis and category development. The fourth author is an associate professor at a research-intensive institution in the Midwest and has expertise in Black women’s overall health. She served as expert auditor and assisted with category development. The fifth author is a doctoral student and is the research assistant of the first author. She assisted with the literature review and reviewing the final draft of the paper. Last, the sixth author is a full professor at a research institution in the South. She assisted with data analysis and structure of the introduction and literature review.

Because all authors identified as middle-class Black women, we discussed throughout the research process how our biases, assumptions, and identities might influence our interpretations of the findings. We believed most middle-class Black women would discuss what it meant to them to be well through a race, class, and gender lens. We expected many women would discuss wellness specifically as it related to their weight. We also believed some women would discuss their conceptualizations of wellness in relation to their spiritual practices and beliefs. Lastly, given the intersectional framework used to guide this study, we expected women to discuss wellness in a way that did not align with traditional ways wellness has been examined (i.e., biopsychosocial; six dimensions of wellness; biopsychosocial-spiritual).

### Semi-structured interview and procedures

In-depth semi-structured interviews were used to explore middle-class Black women’s experiences with wellness. The in-depth nature of the interviews allowed us to assess intersectional factors associated with the lived experiences of wellness among middle-class Black women. First, women were asked to select a pseudonym, which was used throughout the data collection and analysis processes. Second, women were asked to define wellness. Third, women were asked to describe their understanding of what it meant to be emotionally and mentally healthy. Lastly, participants were asked to describe their experiences with wellness in the context of who they are. Experiences with wellness emerged as participants described how they saw themselves in relation to the context in which they lived. Follow-up questions explored participants’ wellness practices and examples of times when they knew they were and were not well. As participants shared examples, probing questions were used as needed. Interviews ranged from 60 minutes to 5 hours, but on average, lasted 2.5 hours. Interviews were completed in participants’ homes, coffee shops, or mutually agreed upon locations. Interviews were audio recorded and transcribed verbatim by a professional transcription service and imported into Dedoose Version 9.0.17 (2021) for data management and analysis.

### Constructivist grounded theory data analysis

Grounded theory allows researchers to investigate participants’ experiences to develop a new social process or concepts that need further exploration (Charmaz, 2014; Charmaz & Thornberg, 2021). The constructivist approach assumes social reality is constructed, multiple, and processual (Charmaz, 2014). Understanding the specific conditions of the constructivist approach of grounded theory—social, cultural, and other contextual factors—acknowledges the women as true experts of their realities in the research process and captures the hidden hierarchies of power associated with their experiences of wellness (Charmaz, 2006, 2014).

As outlined by Charmaz’s constructivist approach to grounded theory, data analysis occurred simultaneously with data collection (Charmaz, 2006, 2014). First, the first author read each transcript individually and wrote analytic memos to capture an understanding of each interview. Second, the first author conducted initial coding, consisting of line-by-line coding to mine data for analytic ideas to examine in future data analysis (Charmaz, 2006, 2014). In this context, the first author focused on answering questions about the data to gain a deeper understanding of the data, what the data suggest, and from whose point of view (Glaser, 1978; Glaser & Strauss, 1967). Third, the first author conducted focused coding, which was more selective (Charmaz, 2006, 2014). This selective phase of the coding process required the first author to sort and synthesize the most significant initial codes to advance the theoretical direction of the initial coding process and the conceptual depth of the emerging theory (Charmaz, 2006, 2014). Fourth, the first author conducted axial coding to build properties and dimensions of the categories of the emerging grounded theory (Charmaz, 2006, 2014; Strauss & Corbin, 1998). During this time, the first author started making theoretical connections across each transcript. To capture these theoretical connections, the first author developed various diagrams of the data to illustrate associations between emerging categories (Charmaz, 2006, 2014; Strauss & Corbin, 1998). The categories that formed were the groupings we placed on the various codes to continue to deepen our analysis (Charmaz, 2014). Fifth, the first author engaged in theoretical coding to help examine the relationship between the core concept of mind, body, and spirit with the other codes and concepts emerging from the theory. Theoretical codes were developed by the first author and verified by co-authors to ensure a clear understanding of the wellness phenomena among middle-class Black women. Examples of theoretical codes used were strategies, engagement, wellness, and context. The coding and analysis process was iterative and continued until saturation was reached with each concept and category (Charmaz, 2006).

### Rigor and quality

For a constructivist grounded theory study, Charmaz (2006, 2014) outlined four main criteria to ensure quality: credibility, originality, resonance, and usefulness. Credibility focuses on making sure data are compared systematically and considers researchers’ views and actions (Charmaz, 2014; Charmaz & Thornberg, 2021). In alignment with the constructivist approach to grounded theory and credibility, we remained reflexive throughout the research process. We asked questions of the data to assist with collecting data that would help us better understand how culture and community influence middle-class Black women’s perspectives of wellness. For instance, we considered the multiple ways middle-class Black women discussed wellness and were often challenged by how middle-class Black women discussed their experiences with wellness in relation to women of other racial and ethnic groups.

Another way to ensure quality in constructivist grounded theory studies is through resonance, which “demonstrates that the researchers have constructed concepts that not only represent their research participants’ experience, but also provide insight to others” (Charmaz & Thornberg, 2021, p. 12). As we realized wellness for middle-class Black women was about a holistic concept they worked to practice, we gained resonance by hearing the women’s narratives from a fresh standpoint and revised our interview questions. New codes emerged to understand the women’s behaviours and feelings about wellness and helped us construct the concept of stressing. Stressing highlighted how factors like time and other responsibilities influenced their holistic approach to wellness. We theorized how middle-class Black women managed their stressors and influenced their experiences with wellness. Usefulness, like confirmability in qualitative research, helps researchers clarify the stories participants share about their lives (Charmaz, 2014; Charmaz & Thornberg, 2021; Lincoln & Guba, 1985). To achieve openness, we asked clarifying questions to help us understand what it meant for the middle-class Black women in this study to be well.

We also used several techniques to enhance credibility and trustworthiness of the study, including multiple coders, memos, and rich quotes. Multiple coders were used to establish consistency in applying codes (Hill et al., 1997; O’Connor & Joffe, 2020). Memos served as audit trails to document the process and product such as findings, interpretations, field notes, and coding themes used in the data analysis process (Lincoln & Guba, 1985). Our memos included detailed information about our codes and outlined remaining questions, all of which raised the analytical level of our theory development (Charmaz, 2014; Charmaz & Thornberg, 2021). To further deepen our theory development, we used all relevant data from the interviews to reach theoretical saturation, as no new properties of the categories were emerging (Charmaz, 2014).

Last, to enhance credibility and trustworthiness of the study, we discussed how some scholars may argue that individual interviews that range from 60 minutes to 5 hours, but on average lasted 2.5 hours can seem unusual. We also discussed how other scholars posited that “qualitative interviews take many forms and are used in many different contexts for a wide variety of purposes (Irvine, 2011, p. 204). As such, during each individual interview the principal investigator (PI; first author) checked in with each participant to make sure there were no concerns given the content being explored and the time (e.g., length) of their interview. None of the participants ended the interviews early nor did they report any concerns regarding the content being discussed. Finally, we made sure we analysed the data that was most relevant for this study and when discrepancies arose, we discussed the discrepancies until we reached consensus.

### Ethical considerations

Prior to the recruitment of participants, the Institutional Review Board (IRB), IRB Committee 2 at the University of Houston (STUDY00000782) approved this research. Additionally, all participants gave both written and verbal consent before participating in this study. Although the study focused on middle-class Black women’s experiences with wellness, the principal investigator (PI) of the study debriefed with each participant before and after each interview to ensure their well-being. It should be noted that none of the participants expressed adverse experiences from the interviews.

## Results

**Figure 1.**
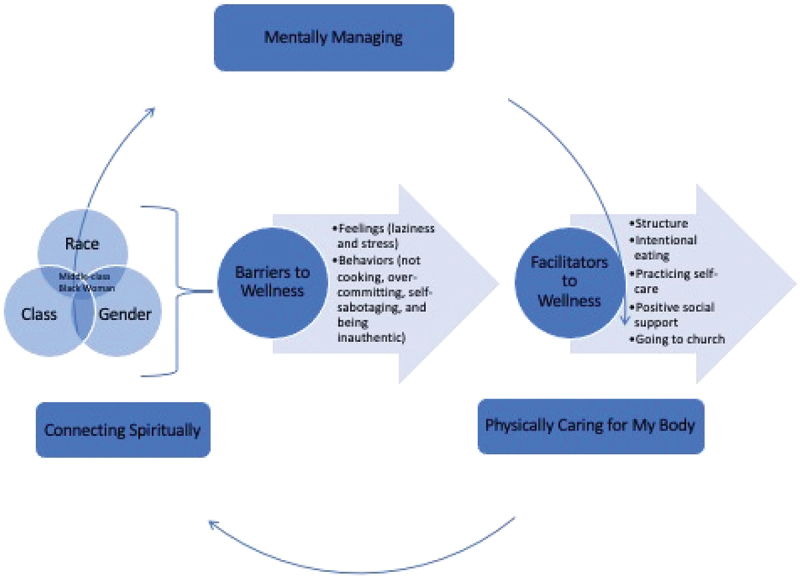
Model of wellness among middle-class Black women.

Among Black middle-class women in this study, the core category included the following: (a) mentally managing, (b) physically caring for my body, and (c) connecting spiritually. The two main categories that emerged and informed how the women experienced wellness were (a) barriers to wellness and (b) facilitators of wellness. Each category consisted of subcategories (see Figure 1). The sections discussed below are an illustration of how middle-class Black women conceptualized their experiences with wellness, the categories that influenced their wellness experiences, as well as the barriers and facilitators they encountered when trying to maintain their sense of wellness.

### Mind, body, and spirit: core category

The core category of wellness among the Black middle-class women in this study was mind, body, and spirit. Mind, body, and spirit was situated in the women’s narratives with wellness that centred their identities as Black middle-class women. Likewise, mind, body, and spirit focused on an integration of and a balance between the women’s mental, physical, and spiritual wellness. Thus, the women described their core experiences with wellness as (a) mentally managing, (b) physically caring for my body, and (c) connecting spiritually.

#### Mentally managing

For mind, body, and spirit, mentally managing reflected the importance of having balance in one’s life as a middle-class Black woman and it focused on their mental well-being. The majority of the women in our study reported on the importance of being able to manage their mental health as a way for them to be well because they had to take care of everything. Having to take care of everything for everyone as women who have “somewhat made it” caused pressure and made it hard to manage mentally. The women often felt like they were constantly trying to manage mentally at the expense of themselves, so finding balance was elusive at times and stable at other times. For one woman, the pressure she felt developed as a result of her feeling the pressure to be everything for her family. For example, Emily shared:

There’s this pressure, no matter what class you are, as an African American woman. Maybe especially as one who has somewhat made it, because you are middle-class, to uphold the family every which way. Financially, emotionally, we take care of everything. So, the only thing you can do is just manage emotionally and find some kind of balance because you’re too tired to do anything else.

Additionally, Jade mentioned that she felt the pressure and felt like she struggled to manage mentally because her dad was sick:

Like, when my dad passed away five years ago from cancer, I struggled a lot mentally and with everything. When my dad was sick, there was no balance, because when I got off work, I went home and took care of my daddy. There are times and seasons where you don’t get to do that, but in the times when we can, we have to allow ourselves to do that and be okay with that despite the stress and pressure you may feel. My grandma said, “You doing a whole lot and ain’t doing none of it well.” There’s no way that you can give. There’s only one you. There’s no way that you can give 100% to everything.

Jade went on to share that for her managing mentally was about having balance and prioritizing. She shared:

For me, balance has been very important. But we Black women with some money and a little bit and I mean a little bit of time in our schedule, I think, don’t balance. We don’t prioritize … And then you’re struggling, out of balance, and mentally barely making it.

Ultimately, for Emily and Jade, the pressure they experienced from their family was critical to them having balance and influenced how they managed mentally. Further, Emily’s and Jade’s statements draw attention to what other women shared about what it meant to have money and some time that could help or hinder their ability to balance in order to manage mentally because of the expectations family had for them to help. The data from the interviews had several parallel examples of the women situating their experiences with wellness in the context of finding balance between their mind, body, and spirit given the pressures they endured specifically from family.

#### Physically caring for my body

For the middle-class Black women in this study, physically caring for their body also influenced their experiences with wellness. In fact, the need to physically care for their body was connected to their need to feel balance in their lives physically. Women had varying responses in how they physically cared for their body. However, most of the women shared that physically caring for their body was about engaging in physical activities like yoga and running. For Indigo, she intentionally chose to participate in yoga as a practice for balance and to physically care for her body. When she discussed why she practiced yoga she noted that she wanted balance to help her manage physically and mentally.

I love yoga … because we [Black women] don’t tend to take care of our self in that way so doing yoga and maintaining a yoga practice helps me to take better care of myself, helps feel like I’m balancing the stress I feel mentally. And it’s like I want to feel like my body is in sync with my mind when things become too much.

In addition to yoga, several women noted running was another way they took care for their bodies physically because they needed to find balance with their bodies. For instance, Afro stated:

I started running in the last 2 years because I needed to and not a lot of Black women were doing it. I ain’t like a fast runner, nothing like that. But you know what? I’m glad I’m doing it because it gives me a sense of balance with the rest of my body. My story was always, I have asthma, I can’t run. But yeah, I run to stay active and physically and mentally healthy. That’s really what’s driving it for me. I think, too, wellness is very much mental and as a Black woman who is a professional, I have a lot on my plate that I have to tend to.

Similarly, Katie explained that she began running because she needed to figure out what to do to find balance in her life.

I think we do, many of us, the majority of us see that it’s healthy because I can see it on my Facebook feeds. People running marathons. People getting fit. People working out, during lunch, just trying to figure out a way to do it to find balance in my life. I definitely think there’s a movement that reminds us of that. I used to say that in 2000. When you saw the White girl running on the lake in 30-degree weather, that is her drug. That is like she gotta do that. That ain’t that she loves running. She knows what happens when she doesn’t do that. So I think we’re getting there.

Even though most women discussed the importance of physically caring for their bodies and identified activities they enjoy engaging in, one woman shared how she struggled to physically care for her body. When asked what it meant for a middle-class Black woman to be physically well, she said, “Oh boy, I don’t know … Because I’m only 30 and I already know that I’m tired. I have grey hair, a lot.” (Daphne, 30)

#### Connecting spiritually

Women reported that connecting spiritually (attending church and praying) during hard times was an important aspect of how they conceptualized their experiences with wellness. For one woman, connecting spiritually meant tending to the many challenging things in her life through the church to feel well. Lisa stated:

I think that spiritual piece holds more weight in the Black community just because of the history of our race, especially in the United States, a lot of it is connected to religion and our faith. And so, we make it through things. We deal with things centered through the church and the things that we learn in the church, and so that’s why I think those things [attending church and praying] are really important for us to feel well.

Echoing Lisa’s sentiments, Shelia discussed how connecting spiritually was a way for her to stay out of a dark place:

So, I grew up in the church, but it’s only been when I was in college that I really started having a better relationship and was really applying the Bible to my life … yes, my spiritual walk has made a tremendous impact on my life … so I try my absolute best to stay focused to stay encouraged, to stay positive. I think that’s very key to just be positive. Because being negative sucks all the energy out of you. So, yeah, definitely my whole spiritual life—going to church, praying, reading my bible—has been tremendous in keeping me away from that dark place and is an important part of my wellness practice.

The need to recite bible verses was something Shelia was taught at a young age that Black women did especially when times were challenging. Thus, the need to be connected spiritually in times of challenge encourage Black women to rely on practices they were taught to help them feel balanced. In fact, Michelle shared similar comments:

I would say that praying is strong within the Black community and has been for years. So, I think a lot of prayer comes into play. What you tell yourself about a situation plays into the emotional side of being able to manage the stress and possibly depression. If you have whatever faith that is—Christians say I can do all things through Christ who strengthens me [Philippians 4:13] that they say all the time to themselves. There’s so many, a whole host of different bible verses that you can use to encourage you or to get you out of the funk. Have you think a little differently about your situation … . you have a mantra or scripture or whatever, then it helps you get out of that. Then you’re not into them. I think that helps your emotional state as well. It gives you a piece of mind.

In sum, mind, body, and spirit was about the women’s need to seek balance in their lives emotionally, physically, and spiritually. Yet, despite their desires for finding balance between their mind, body, and spirit, the women also reported specific barriers and facilitators towards being well.

### Category 1: barriers to wellness

Middle-class Black women discussed various barriers they faced for them to be well. In fact, the barriers the women identified hindered them from being well was illuminated through a wide range of (a) behaviours (e.g., over-committing and self-sabotaging) and (b) feelings (e.g., laziness, stress, and inauthenticity).

#### Behaviours

Although women reported a wide range of feelings that presented as barriers to them being well, the primary behaviours some of the women reported that got in their way of being well were over-committing and self-sabotaging. Jade shared:

All these obligations and all these things. We [Black women] overcommit ourselves. So that is why we struggle with being well. Not like we don’t want to be well, we just do too much that makes it hard to be well or do anything to be well when we over commit.

Other women also reported similar behaviours but talked about over-committing and self-sabotaging as getting in their own way. These women felt as if getting in their own way was such a common occurrence for middle-class Black women, they sometimes did not recognize what they were doing. Amber discussed:

I think we [Black women] get in our way. I think a lot of it is unknowingly … . not like we’re trying to self-sabotage. I just think we don’t know a lot of the time. I think the demands and obligations of the world: job, family, and all of those things get in the way.

#### Feelings

Feelings such as laziness, stress, and inauthenticity were often reported by the women as the primary barriers to them being well. For instance, Queen shared:

Just laziness. Just laziness. Do I want to go upstairs and make a smoothie or do I want to numb out and watch Love and Hip Hop [a television show]? I want to numb out because I can’t process things right now.

Valerie noted cooking was a form of stress and served as a barrier to her being well, reporting:

I have to say just for physical stuff and the stress of cooking, I would imagine at this age many of us have not been taught to cook in a healthy way … . My mother didn’t cook soul food growing up, but it wasn’t super healthy food … So even the stuff I cook now, I have to think about it. I’m like wait, what are we putting in there. I’m not putting in a stick of butter.

For other women being inauthentic was a barrier to them not being well. When the women were not authentic, they felt like they were wearing a mask or not honouring themselves which compromised their wellness. In the following quote, Lola shared how being authentic meant she no longer had to wear a mask, describing:

As I reflect on what it means to be authentic. I have to think about a time when I was broken … . We really try to cover it up, try to mask it … I’m a strong Black woman. I have to do this. I have to show them that I can do this. I still have to put on my mask. I put it on sometimes because sometimes I need to get through my day and I don’t want to get people asking me what’s wrong when it’s maybe just me pondering something … . But now I feel like I’m able to put my mask on in a healthy way … . How I present myself in certain dynamics, in certain company, or formal events versus an informal one, yes, absolutely I still think about that. But I am more authentic and being authentic when I’m making decisions about me and what I want to do.

Like Lola, another woman discussed how being authentic meant she was being true herself. Afro stated:

I think for me it’s are you being true to yourself and what do you allow in your space and how are you really being authentic as well? I think that’s important. That’s something that I try to work on every day.

Essie echoed Afro’s sentiments. She noted that being authentic made her explore what was important to her: “It’s like finding what’s important to you—you know, like that authentic you and who you are.” Additionally, the barriers to wellness many women expressed were shaped by their need to release pressure from the stressors many middle-class Black women experience in their lives. Amber described how important it was for middle-class Black women to just be themselves as a way to release pressure.

I think just feeling like you could be yourself, because I think a lot of times you can be looking around like who is this person? Adjusting to being so many different things to so many different people, and so many different situations, you lose the essence of who you are … . I think that if we can be our true, authentic selves, that would take a lot of pressure off of us.

In this quote, Amber mentions the multiple roles Black women who are middle-class take on for other people and feel like she cannot be herself, which causes a lot of pressure. The pressure from being inauthentic in many roles in their lives may occur due to Black women’s experiences of having to be the caretaker for others including those in their family especially if they have money. The extra burden they may feel as middle-class Black women to take care of others even when they do not want to can negatively impact their ability to be well because they would be too tired to care for themselves. Although many Black middle-class women in this study rarely had any disagreement with the barriers they experienced to being well, most of the women appeared to have had ah ha moments when discussing the barriers to their own wellness conceptualizations. In fact, the women collectively agreed that they have never had the opportunity to think about what hinders them from being well even though they tried.

### Category 2: facilitators to wellness

The women identified five primary ways that facilitated their wellness practices: (a) planning/structure, (b) intentional eating, (c) practicing self-care, (d) having positive social support, and (e) going to church.

#### Structure

Several women noted that having structure was an important facilitator for them to be well. For Danielle, not having structure was frequently linked to her not being well. When she discussed not having structure she highlighted how she did not function well.

For me to be well, and say to be physically and mentally well, I have to have absolute structure. My schedule has to be on point. My meals have to be ready. I have to have in my mind what I’m going to wear. Everything has to be structured so when things are not like that … . I get like flustered and I don’t function well.

Danielle’s need for structure like many other women in this study drew attention to the many tasks they had to balance and manage including caring for children and their partners. For Danielle, maintaining structure also outweighed the consequences (e.g., depression, anxiety) if she did not have structure, as she noted that as a middle-class Black woman “you’re always expected to give an do more because people assume you have it to give.”

#### Intentional eating

Another very important facilitator of wellness for the women in our sample was intentional eating. The intersection of eating well and being a middle-class Black woman resulted in unique perspectives of wellness, as the women highlighted that selecting food with good intentions, eating properly, and being intentional were critical aspects of their path to wellness. Angela shared how intentionally choosing what her body needs is connected to eating well. She noted:

For me, physical wellness means that it requires some intention. It means having food for your body. It means choosing your foods with intention, like thinking about what your body needs. And meeting those needs, nutritionally.

The following women expressed similar sentiments: “Making some choices. Like with our food. I love my food. It has to be a certain way, but moderation in everything.” (Emily) “When I’m eating properly.” Lisa also stated, “it’s also eating right.” (Elaine) “Eating better is important … . thinking about what I’m cooking … So paying attention to my body and eating better for my body is important.” (Sherry) What is important to note with these women about intentional eating as a facilitator of wellness for them is that they had options. Being a middle-class Black woman allowed them the privilege to be selective in their food choices without or with few consequences to their finances.

#### Practicing self-care

Overwhelmingly the women agreed that waking up early and journaling were activities they needed to engage in to support their self-care practices. Practicing self-care meant that the women were making time for themselves and the time they made for themselves was not interrupted for or by anyone else. Like Katie said, “waking up early is a way for her to practice self-care”:

I think it means feeling really, really good from the inside out. For me, and I tell my husband all the time. I’m like, “Ooh, I love when I wake up early before it’s time to get up” because those are my hours of 5:00, 6:00, in the morning … I can journal, something I love to do. I can kind of have my day, but I’m greeting the day.

This quote shows the importance of the need for middle-class Black women to centre their own needs without others being around because when others are around their attention shift to what others need and want. Thus, Katie’s desire to wake up early to practice self-care was critical for well-being and outweighed her need to care for others before herself, something Katie said, “Black women with a little bit of money do all the time … take care of other folks.”

Self-care was also influenced by having the financial means to change the scenery if you needed to. Monique described how changing the scenery and changing things up was important to her self-care practices:

You know as Black women with degrees it’s important for us to make sur we are okay … So if that means you’ve got to get some new scenery, and change it up, then you need to do that. If that means you need to go and talk about it, to get it taken care of, that’s cool. But you have to do something to make sure you’re taking care of yourself. You know.

In this quote, Monique refers to the financial benefit of being a middle-class Black woman by noting how because she has a degree—an assumption that means she has money to engage in self-care practices—she can travel to take care of herself. The ability to travel without question to their finances is one way in which middle-class Black women self-care practices may differ than Black women with less income. Additionally, their self-care practices highlighted an important aspect of time and how many middle-class Black women in this study referenced the flexibility they had in their schedules to practice self-care because of the different professional, managerial, technical, or administrative job they had.

#### Positive social support

For all women in this study, positive social support was critical to their wellness experiences. Further, positive social support focused on close relationships with their girlfriends and going to therapy when needed. In fact, several women described the importance of their girlfriends in how they understood their experiences with wellness. Although her connections were through social media, for one woman, she always felt better because of the social support she got from her “sistah friends.” For instance, Kendra said, “But I think that now I feel more community. And it’s probably because of social media, I think. Because I can have access to a lot of my sistah friends for support.”

In a similar fashion, Indigo discussed how her girlfriends were a positive source of support for her because they were people who loved her and who were good to her. She shared, “I think wellness is about surrounding myself with people that love me and are good to me, even if I don’t always feel like I want to connect with them.” Katie also discussed how her social support came from a particular girlfriend, comparing her relationship with this specific friend as an accountability partner because they “just knew each other’s looks.” She said:

We’re accountability partners, and we do a lot of talking about it [i.e., challenges they experience in their lives]. But my accountability partner, she’s my college roommate and she lives in New York … . after we started talking about it, and the look in her eyes, I just—I was like, I know that look. It’s a weird look, but I know that look. I said, “Yeah, we’re going through the same things.”

What these quotes collective note is that many middle-class Black women in this study reported the importance of close relationships they had with girlfriends since college and the new relationships some of them developed while in their current professional roles. What was also highlighted in these friendships were an assumed shared understanding about what it meant for them to be middle-class Black women given their personal and professional environments, as they did not have to explain who they were when they were talking with their girlfriends.

Lastly, for some women, other forms of social support included going to therapy. In fact, some of the women reported therapy could help sort out some struggles they were experiencing when girlfriends could not, as therapy was a form of social support that was seen as maintenance. For example, Sherry discussed:

One thing I did say was that I would love to go see a therapist, if for nothing else because you need that outlet. The same way you go to the dentist for maintenance. I don’t go see my dentist when my tooth is falling out. It’s annual maintenance and care. Even in that situation I was like man, I’m not in touch as I used to be but it’d be great to have someone. Even going into a marriage, I would go for marriage counseling. Not that we’re having issues but just because you know that it helps. It’s another form of social support that we do not consider often.

In this quote, Sherry posits that going to therapy was a form of care and support when she needed it. Although studies have highlighted that very few Black women go to therapy (Nelson et al., 2020; Walton & Oyewuwo-Gassikia, 2017), when they do go to therapy, they feel better and feel a sense of positive social support like Sherry. The need to feel a sense of positive social support for middle-class Black women when they go to therapy is vitally important as it allows many of them to be themselves without judgement, expectations, or centring other peoples’ needs.

## Discussion

The purpose of this constructivist, grounded theory study was to investigate how middle-class Black women experience wellness through an intersectional lens. We found middle-class Black women’s experiences with wellness were experienced within the contexts of their race, class, and gender identities. Women described their experiences with wellness as seeking balance between their mind, body, and spirit. Most of the women shared experiences that highlighted their desire for wellness but noted the barriers and facilitators they faced with trying to be well as middle-class Black women. The women’s lived experiences as middle-class Black women in the United States may contribute to the unique ways that influence their conceptualizations of wellness. Given that the current models of wellness do not include key cultural or contextual factors that contribute to wellness, this study contributes to the literature by examining how race, class, and gender intersect to influence middle-class Black women’s unique experiences with wellness. As such, we created a unique model for understanding wellness among middle-class Black women.

Although, our model includes factors that may seem to be well known and commonly proposed suggestions to wellness for Black women in general and possibly other women of different racial and ethnic backgrounds, what was unique about the middle-class Black women in this study is that they discussed how the pressures from their family created influenced many of their wellness practices. Like middle-class Black women in previous studies (Howell, 2023; Knighton et al., 2022; Sacks, 2019), the women had stressors such as family needs that influenced their wellness experiences. To be well, the middle-class Black women felt they had to manage mentally, physically, and spiritually early in the morning for them to not have any interruptions. As a result, middle-class Black women often had to figure out how to make time for themselves or struggle with managing everything. Additionally, research has shown that middle-class Black women intersectional identities of race, class, and gender can influence their lived experiences with different tropes—Strong Black Woman, Superwoman Schema—about Black women (Knighton et al., 2022). Middle-class Black women should not have to negotiate key relationships (i.e., family) or act as superhumans in order to make time for themselves so they can have balance mentally, physically, and spiritually.

We found that managing mentally was a significant category related to middle-class Black women’s experiences with wellness. Previous research shows that managing mentally is often a common practice for Black women that adversely impacts their health (Beauboeuf-Lafontant, 2007; Woods-Giscombé, 2010). In fact, managing mentally shows up in Black women as the Strong Black Woman (Beauboeuf-Lafontant, 2007) or Superwoman Schema (Woods-Giscombé, 2010). Unfortunately, the consequences for middle-class Black women having to manage mentally is exhausting and has a significant impact on their mental health (depression, anxiety, increased stress; Bell, 2017). Future research should examine other ways that middle-class Black women manage mentally that highlight their strength and resilience.

Our findings illuminate how physically caring for their bodies was solely about intentional eating and exercising. Intentional eating or some of the women was a way for them to find balance. The desire to find balance through intentional eating may reflect the intersection of privilege and oppression as it relates to the race and class dynamics many middle-class Black women in this study may contend with given their middle-class standing. Ideas about what it means to intentionally eat dovetailed the women’s ideas about exercising. Our findings demonstrate that exercising is important yet many of the activities the women in this study enjoyed (yoga and running) they did not see a lot of Black women doing. Ideas about exercising among middle-class Black women may also reflect the intersection of race, class, and gender beliefs about exercising and the types of exercising Black women within the United States should and should not engage in. Although previous research has highlighted barriers to middle-class Black women’s physical wellness practices, such as time allocation, racial composition of neighbourhoods, and body image (Ray, 2014), a recent systematic review have found that when Black women have engaged in physical activities they were more confident in their body and their overall well-being improved (Obi et al., 2023). Our findings highlight the need for wellness interventions to specifically address the intersectional identities among middle-class Black women and the need for understanding both the privileges and oppressions they experience with being well. One strategy to address the various ideas that influenced the eating and exercising middle-class Black women engaged in is to unpack the cultural messaging and norms the women learned about eating and exercising.

For women in our study, connecting spiritually was an important aspect of their need for balance between their mind, body, and spirit. Several women shared they needed to have balance mentally and physically in order to address the stressors they endured in their daily lives. One woman shared how praying helped her find connection spiritually, but also mentally and physically. This not only illuminates the importance of balance in the lives of middle-class Black women who are harmed by gendered racism and classism, but also speaks to the ways in which middle-class Black women are willing to work towards centring their needs and shifting a deficit approach to a wellness approach to improve their overall health and find balance with their mind, body, and spirit. Although previous research shows that Black women are sicker and disproportionately face poorer health outcomes (Chinn et al., 2021; Sacks, 2019; Williams, 2002), the middle-class Black women in this study were engaging in spiritual (praying and going to church) practices that have been in the Black community for decades to help them find balance (Chinn et al., 2021; Mattis, 2002; Woods-Giscombé, 2010).

In addition to the middle-class Black women in this study seeking balance with their mind, body, and spirit in relation to their experiences with wellness, they also identified barriers and facilitators to their experiences with wellness. Prior research on Black women’s experiences with wellness draws our attention to the ways in which Black women desires for wellness are within the context of the physical and mental health challenges they were experiencing (Chinn et al., 2021; Donovan & West, 2015; Jones, 2004; Jones et al., 2014; Nicolaidis et al., 2013; Waite & Killian, 2008; Woods-Giscombé & Black, 2010). In fact, Chinn and colleagues (Chinn et al., 2021) posit that the health of Black women is measured by their poor health outcomes and is a direct result of the barriers they experience in receiving quality health care, racism, and stress associated with the unique experiences of Black womanhood in U.S. society. Thus, the middle-class Black women in our study focused on key aspects of wellness that pushes us to think about wellness more holistically and that includes their mind, body, and spirit. Our findings echo other studies (Bell, 2017; Black Women’s Health Imperative, 2019; Jones et al., 2015) that suggest a holistic approach—emotional, physical, and spiritual—to wellness for Black women was critical for their success in mental health or substance abuse treatment, as well as in finding balance in their daily lives.

Like previous findings, we found that staying connected spiritually through knowing the history of the significance of the church in the Black community, attending church, and praying were important components of middle-class Black women’s conceptualizations of wellness. Spirituality and religious practices are historically rooted within the Black community, regardless of one’s socioeconomic position. In fact, scholars have noted that the use of prayer, meditation, and church attendance serve as tools for coping with stressful situations (Bacchus & Holley, 2005; Everett et al., 2010; Mattis, 2002; Reed & Neville, 2013). Our findings echo previous research and demonstrate the importance of middle-class Black women staying connected spiritually to manage stressful situations. In their qualitative study, Everett and colleagues (Everett et al., 2010) noted that during times of stress, Black women often rely on family, friends, prayer, and attending church to help them manage.

## Practice implications

We provided several important implications for practitioners working with Black middle-class women when examining their conceptualizations of wellness. First, it is important for practitioners working with middle-class Black women to understand their experiences with wellness. In doing so, practitioners can gain a deeper understanding of how middle-class Black women’s need for connection and balance between their mind, body, and spirit could support or hinder their ability to be well. In this context, practitioners can create safe spaces for Black middle-class women to be vulnerable as well as support and encourage them to develop a plan to intentionally address factors that can support their journey towards wellness while simultaneously identifying the barriers they may experience to their wellness. Further, practitioners can use assessments that centre the whole person, such as the biopsychosocial-spiritual model. Lastly, practitioners can develop treatment plans that can cultivate ways to become well such as self-care or intuitive eating.

Second, for middle-class Black women, practicing from an intersectional lens is critical to gain a deeper understanding of the multiple contextual factors influencing their physical and mental health outcomes (Lewis et al., 2017). Third, we recognize social support is an important aspect of their wellness practices; the women in this study were open to going to therapy as a form of maintenance, but it was not seen necessarily as the first nor only way to manage emotionally. It may be helpful for clinicians working with Black women in general and middle-class Black women more specifically to consider using sister circles as a possible intervention for Black women (Neal-Barnett et al., 2011). Fourth, we hope practitioners think about ways to practice intentionally when working with middle-class Black women on a journey towards wellness. Practicing with intentionality pushes clinicians to see women as experts of their lives, to create collaborative relationships. It places the clinicians in a position where they are learning from the women with whom they are working (Walton & Boone, 2019). Last, we encourage practitioners to attune to a model of wellness that supports middle-class Black women’s desires to be well. For the Black women in this study, the connection between their mind, body, and spirit was essential to being well.

## Limitations and future directions

The results of this study should be tempered considering several limitations. First, the women of this study primarily resided in a large Midwestern city in the United States, which limited our understanding of wellness among other women in different geographical locations. Future research should consider wellness experiences among middle-class Black women in other geographical locations, as well as with Black women in rural communities. Second, there is little agreement on the definition of middle-class in the literature. However, all of the women in this study identified as middle-class, and the majority of these women (63%) also grew up in middle-class families. It is not surprising that many of these women discussed ways they experienced wellness and ways they have experienced barriers in relation to their wellness practices, as many middle-class Black women report some self-care practices despite the many barriers they experience (Ray, 2014). This is not to say that women who have lower incomes do not have any wellness practices. It is plausible that women with lower incomes have similar wellness practices as the women in this study; further investigation is needed to learn about the ways low-income Black women experience wellness. Lastly, for this study, we focused only on middle-class Black women between the ages of 30 and 45. Future researchers should consider the experiences of wellness among middle-class Black men, because a growing number of Black men have moved into the middle class (Institute for Family Studies, 2018; Wilcox et al., 2018). Lastly, it would be helpful for future research to longitudinally explore wellness experiences with Black women to gain a better understanding of how their wellness experiences may change over time and how they may resolve some of the barriers they endure along their wellness journey.

## Conclusion

Wellness is an understudied topic among Black women, specifically middle-class Black women. We hope practitioners, researchers, and other affiliated providers who work with middle-class Black women examine what is going right, such as their conceptualizations of wellness, instead of exploring what is wrong. Recognizing the possibilities among middle-class Black women can begin to shift the narrative from one of a deficit to one of strength and empowerment. By identifying middle-class Black women’s conceptualizations of wellness and their wellness practices, clinicians can work with middle-class Black women on addressing challenges they present in therapy and use key contextual factors to ensure their wellness.
